# Estimating the Risk of Severe Peanut Allergy Using Clinical Background and IgE Sensitization Profiles

**DOI:** 10.3389/falgy.2021.670789

**Published:** 2021-06-07

**Authors:** Mareen R. Datema, Sarah A. Lyons, Montserrat Fernández-Rivas, Barbara Ballmer-Weber, André C. Knulst, Riccardo Asero, Laura Barreales, Simona Belohlavkova, Frédéric de Blay, Michael Clausen, Ruta Dubakiene, Cristina Fernández-Perez, Philipp Fritsche, David Gislason, Karin Hoffmann-Sommergruber, Monika Jedrzejczak-Czechowicz, Laurian Jongejan, Marek L. Kowalski, Tanya Z. Kralimarkova, Jonas Lidholm, Nikolaos G. Papadopoulos, Todor A. Popov, Nayade del Prado, Ashok Purohit, Isabel Reig, Suranjith L. Seneviratne, Athanassios Sinaniotis, Emilia Vassilopoulou, Serge A. Versteeg, Stefan Vieths, Paco M. J. Welsing, E. N. Clare Mills, Thuy-My Le, Aeilko H. Zwinderman, Ronald van Ree

**Affiliations:** ^1^Department of Experimental Immunology, Amsterdam University Medical Center, Amsterdam, Netherlands; ^2^Department of Clinical Epidemiology, Biostatistics, and Bioinformatics, Amsterdam University Medical Center, Amsterdam, Netherlands; ^3^Department of Dermatology and Allergology, University Medical Center Utrecht, Utrecht University, Utrecht, Netherlands; ^4^Allergy Department, Hospital Clinico San Carlos, Instituto de Investigacion Sanitario San Carlos, Madrid, Spain; ^5^Allergy Unit, Department of Dermatology, University Hospital, Zurich, Switzerland; ^6^Faculty of Medicine, University of Zurich, Zurich, Switzerland; ^7^Clinic for Dermatology and Allergology, Kantonsspital St. Gallen, St. Gallen, Switzerland; ^8^Ambulatorio di Allergologia, Clinica San Carlo, Paderno Dugnano, Italy; ^9^Clinical Epidemiology Unit, Preventive Medicine Department, Hospital Clinico San Carlos, Instituto de Investigacion Sanitario San Carlos, Madrid, Spain; ^10^Department of Allergology and Immunology, Faculty of Medicine in Pilsen, Charles University, Prague, Czechia; ^11^Allergy Division, Chest Disease Department, Strasbourg University Hospital, Strasbourg, France; ^12^Faculty of Medicine, Landspitali University Hospital, University of Iceland, Reykjavik, Iceland; ^13^Medical Faculty, Vilnius University, Vilnius, Lithuania; ^14^Department of Pathophysiology and Allergy Research, Medical University of Vienna, Vienna, Austria; ^15^Department of Immunology, Rheumatology and Allergy, Faculty of Medicine, Medical University of Lodz, Lodz, Poland; ^16^Clinical Center of Allergology, Medical University of Sofia, Sofia, Bulgaria; ^17^Thermo Fisher Scientific, Uppsala, Sweden; ^18^Allergy Department, 2nd Pediatric Clinic, University of Athens, Athens, Greece; ^19^Division of Infection, Immunity & Respiratory Medicine, University of Manchester, Manchester, United Kingdom; ^20^Clinic of Occupational Diseases, University Hospital Sv. Ivan Rilski, Sofia, Bulgaria; ^21^Institute of Immunity and Transplantation, University College London, London, United Kingdom; ^22^Department of Nutritional Sciences and Dietetics, International Hellenic University, Thessaloniki, Greece; ^23^Paul-Ehrlich-Institut, Federal Institute for Vaccines and Biomedicines, Langen, Germany; ^24^Division of Internal Medicine and Dermatology, University Medical Center Utrecht, Utrecht University, Utrecht, Netherlands; ^25^Division of Infection, Immunity and Respiratory Medicine, Manchester Institute of Biotechnology, University of Manchester, Manchester, United Kingdom; ^26^Department of Otorhinolaryngology, Amsterdam University Medical Center, Amsterdam, Netherlands

**Keywords:** EuroPrevall, iFAAM, peanut allergy, severity, prediction, clinical background, IgE, component-resolved diagnostics

## Abstract

**Background:** It is not well-understood why symptom severity varies between patients with peanut allergy (PA).

**Objective:** To gain insight into the clinical profile of subjects with mild-to-moderate and severe PA, and investigate individual and collective predictive accuracy of clinical background and IgE to peanut extract and components for PA severity.

**Methods:** Data on demographics, patient history and sensitization at extract and component level of 393 patients with probable PA (symptoms ≤ 2 h + IgE sensitization) from 12 EuroPrevall centers were analyzed. Univariable and penalized multivariable regression analyses were used to evaluate risk factors and biomarkers for severity.

**Results:** Female sex, age at onset of PA, symptoms elicited by skin contact with peanut, family atopy, atopic dermatitis, house dust mite and latex allergy were independently associated with severe PA; birch pollen allergy with mild-to-moderate PA. The cross-validated AUC of all clinical background determinants combined (0.74) was significantly larger than the AUC of tests for sensitization to extract (0.63) or peanut components (0.54–0.64). Although larger skin prick test wheal size, and higher IgE to peanut extract, Ara h 1 and Ara h 2/6, were associated with severe PA, and higher IgE to Ara h 8 with mild-to-moderate PA, addition of these measurements of sensitization to the clinical background model did not significantly improve the AUC.

**Conclusions:** Models combining clinical characteristics and IgE sensitization patterns can help establish the risk of severe reactions for peanut allergic patients, but clinical background determinants are most valuable for predicting severity of probable PA in an individual patient.

## Introduction

Patients with peanut allergy (PA) often require strict elimination diets to prevent potentially severe allergic reactions. Beyond levels of exposure, it is not well-understood why symptom severity varies between patients (1).

To gain insight into severity of PA in a particular patient, accurate clinical evaluation is essential. Besides patient history, routine diagnostic tests include extract-based skin prick testing (SPT) and serum IgE measurements. There is conflicting evidence on the usefulness of SPT and IgE levels for predicting severity of PA (2–5). In recent years, serum IgE testing using whole food extracts has been complemented with allergen component testing. For peanut, IgE to Ara h 2 has been demonstrated to better distinguish PA from tolerance than IgE to peanut extract (6–14). Some studies have reported a relationship between IgE levels to Ara h 2 and severity of PA (7, 11, 14–16), whereas other studies report no clear difference (6, 12, 17, 18). Food challenge, preferably double-blind placebo-controlled food challenge (DBPCFC), is the reference standard for confirming presence and severity of PA. However, due to the burdensome and resource-intensive nature of food challenge, daily practice diagnosis is often based on a suggestive patient history in combination with IgE sensitization (i.e., probable PA) (19).

Peanut and tree nuts are reportedly the most common causes of food-induced anaphylaxis (1). In recent papers on hazelnut allergy (20) and walnut allergy (21), we set out to develop prediction models in which a patient's demographic and clinical background is combined with results from routine extract-based tests and from component-resolved diagnostics (CRD). For both tree nuts, models combining clinical background information with measures of IgE-sensitization were shown to improve the accuracy of predicting severe reactions significantly compared with clinical variables, IgE to extract, or IgE to allergen components alone. Although several previous studies have evaluated the predictive accuracy of combined clinical and serological information for predicting PA (6, 7, 22, 23), the focus is rarely on prediction of *severity*. Petterson et al. developed a model for severe PA based on clinical characteristics and serum IgE against peanut extract, but did not assess contribution of CRD, and included only children (22).

In the present study, we evaluated data collected from predominantly adult patients reporting PA during the EuroPrevall outpatient clinic surveys in 12 different European cities (16), using an approach comparable to that in previous evaluations for hazelnut and walnut. In a subset of these patients that underwent DBPCFC, Ballmer-Weber and colleagues previously reported that systemic reactions occurred significantly more frequently in subjects sensitized to peanut extract (IgE ≥0.35 kU/L) or to Ara h 2 (IgE ≥1.0 kU/L) (16). Our aim was to further investigate the association of demographics, clinical background, and markers of peanut sensitization, with the severity of PA, and to subsequently develop prediction models using all this information to improve discriminatory ability for estimating the risk of severe reactions.

## Methods

### Study Design and Population

Twelve European allergy centers in Athens (Greece), Łódz (Poland), Madrid (Spain), Manchester (United Kingdom), Milan (Italy), Prague (Czech Republic), Reykjavik (Iceland), Sofia (Bulgaria), Strasbourg (France), Utrecht (The Netherlands), Vilnius (Lithuania) and Zürich (Switzerland), enrolled patients with a history of food allergy (FA) in the EuroPrevall outpatient clinic study. Each local ethical committee approved the study. Recruitment took place between 2006 and 2009. Informed consent was documented for all patients before enrollment in the study. For the current study, we included all patients reporting adverse reactions within 2 h of ingestion of peanut.

### Clinical Evaluation

The methodology of the EuroPrevall outpatients study has been described in detail elsewhere (24). All patients underwent an extensive questionnaire, which focused on reaction characteristics and allergic comorbidities, and was administered and interpreted by trained physicians. Skin prick test (SPT) reactivity to peanut extract was assessed using a commercially available extract (ALK-Abelló, Madrid, Spain). Serum samples were collected locally in each center, and analyzed by ImmunoCAP (Thermo Fisher Scientific, Uppsala, Sweden) at the Paul-Ehrlich Institute (Langen, Germany). All available sera were tested for sensitization to peanut extract, as well as to other food and inhalant allergens (24). A custom-made microarray chip, technically resembling the ImmunoCAP ISAC test, was used to test for sensitization to food allergen components, amongst which were peanut allergens nAra h 1 (7S globulin), nAra h 2/6 (2S albumin), nAra h 3 (11S globulin), and rAra h 8 (pathogenesis-related protein family 10 [PR-10] protein) (24, 25). DBPCFC was carried out in all consenting subjects by trained clinicians as described previously (26).

### Definitions

Patients who, along with symptoms within 2 h of peanut ingestion, had IgE sensitization to peanut, as measured by positive SPT, ImmunoCAP or microarray, were defined as having *probable PA*. SPT allergen/histamine wheal ratios were considered positive at a ratio ≥0.5, IgE in ImmunoCAP at levels ≥0.35 kU_A_/L, and IgE in microarray at levels ≥0.3 ISU/L.

Severity of symptoms, as determined from the physician-administered questionnaire, was classified into two groups: *mild-to-moderate* if isolated oral allergy symptoms or symptoms of the skin, eyes, upper airway and/or gastrointestinal system occurred; *severe* in case of symptoms of the lower airway (either laryngeal or bronchial), cardiovascular or neurological system (27, 28). S*kin symptoms* included urticaria, angioedema, erythema/flushing, or itching; *eye symptoms* pertained to conjunctivitis; *upper airway symptoms* pertained to rhinitis; and *gastrointestinal symptoms* included dysphagia, stomach pain, cramps, nausea, vomiting, or diarrhea. L*ower airway symptoms* consisted of throat tightness, dysphonia, dyspnoea, wheezing, cough, or chest tightness; *cardiovascular symptoms* included cardiac arrhythmia, myocardial ischaemia, or hypotension; *neurological symptoms* comprised disorientation/ confusion, dizziness, seizures, incontinence, or loss of consciousness. Severity was based on each participant's most severe reaction to peanut.

Patients with proven sensitization in SPT or ImmunoCAP matching their reported rhinoconjunctivitis or asthma symptoms to birch, grass, mugwort, house dust mite (HDM) or latex were considered to be allergic to the respective allergen sources.

### Statistical Analysis

All analyses were performed in subjects with probable PA. In univariable analysis, differences in demographic factors and clinical background (age, sex, age at onset of PA [<14 *vs*. ≥14 years], symptoms upon skin contact with peanuts [did skin contact with peanut induce adverse reactions, e.g., contact urticaria, dermatitis, rhinoconjunctivitis, bronchospasm or anaphylaxis?], first degree family members with atopy, AD [ever], allergy to pollen, HDM or latex, and sensitization to cats or dogs), results from extract-based testing (SPT and ImmunoCAP with peanut extract), and results from CRD (microarray Ara h 1, 2/6, 3, and 8), were evaluated using chi-square tests, independent sample *t*-tests, or Mann-Whitney *U*-tests where appropriate. Bonferroni corrections were used to correct for multiple testing.

Multivariable analyses were performed to identify the most relevant set of predictors for severity of probable PA. To limit overfitting and improve generalizability, the Least Absolute Shrinkage and Selection Operator (Lasso) regression approach was chosen. This method selects only the most discriminative combination of variables, and applies cross-validation to shrink regression coefficients (29). To ensure use of all data, missing data were imputed ten-fold using the *mice* package in R software. Details on missing data and included covariates are available from Supplementary Table 1. Lasso regression was repeated on each of the 10 datasets. Predictor variables selected in at least 7/10 imputed datasets were included. Bootstrapping was used to estimate 95% confidence intervals (CI) for each coefficient. Results were pooled using Rubin's rules.

A stepwise approach to model building was taken, and the Lasso selection process was applied in each step. In model I, all variables on demographics and clinical background were entered. In model II, peanut extract-based test results (SPT [wheal ratios] and ImmunoCAP [IgE levels]) were added to the selected model I variables. In model III, peanut CRD results were entered, along with the variables selected in model II. Finally, to explore if knowledge of IgE levels to plant source food extracts and components other than peanut could improve prediction of PA severity, ImmunoCAP and CRD results related to sensitization to soybean, lentil, hazelnut, walnut, sesame seed, peach, apple, kiwi, tomato, carrot, and celery, were entered in a final step, after fixing the variables selected in model III. The discriminatory ability of the resulting regression models to distinguish between mild-to-moderate and severe probable PA was quantified by area under the receiving operating curve (AUC) estimators. AUCs were compared using DeLong's test (30).

For comparative purposes, Lasso regression analyses were repeated in a subgroup consisting of only subjects with clinically determined symptom severity based on DBPCFC and subjects excluded from DBPCFC because of a convincing history of severe life-threatening anaphylaxis. The latter subjects were defined as having had a reaction involving hypotension, severe bronchospasm or laryngeal edema within 2 h of peanut ingestion, leading to emergency treatment (24). The principal investigators in Madrid, Utrecht and Zurich reviewed these severe reactions and all agreed upon exclusion of these subjects from DBPCFC, making these patients' history particularly reliable. Subjects with a negative DBPCFC outcome and placebo-reactors were grouped with the mild-to-moderate DBPCFC reactors for this subgroup analysis.

Analyses were conducted with R version 3.4.1.

## Results

Of the 517 subjects reporting symptoms within 2 h of ingestion of peanut, 393 (76%) had probable PA. Overall, 216 (55%) had mild-to-moderate and 177 (45%) had severe probable PA (Table 1, Supplementary Figure 1). Of the subjects with mild-to-moderate probable PA, 89/216 (41%) had isolated oral allergy symptoms (OAS).

**Table 1:** Characteristics of subjects with probable PA.

**Variable**	**Mild-to-moderate (*N* = 216)**	**Severe (*N* = 177)**	***p***
**Demographics**			
Age at visit in years, *mean (±SD)*	28.2 (±14.3)	24.8 (±13.7)	**0.019**
Age <14 years	30/216 (13.9)	39/177 (22.0)	**0.048**
Female sex	126/216 (58.3)	106/177 (59.9)	0.835
**Clinical background**			
Age at onset of symptoms <14 years	86/211 (40.8)	113/174 (64.9)	<0.001^*^
Symptoms upon skin contact with peanut	10/192 (5.2)	48/146 (32.9)	<0.001^*^
Family history of atopic disease	131/210 (62.4)	123/176 (69.9)	0.150
Atopic dermatitis	62/212 (29.2)	89/175 (50.9)	<0.001^*^
Birch pollen allergy^‡^	124/213 (58.2)	81/172 (47.1)	**0.038**
Grass pollen allergy^‡^	124/213 (58.2)	109/172 (63.4)	0.355
Mugwort pollen allergy^‡^	42/213 (19.7)	23/172 (13.4)	0.130
House dust mite allergy^‡^	98/201 (48.8)	106/160 (66.2)	**0.001**
Latex allergy^‡^	10/195 (5.1)	23/165 (13.9)	**0.007**
Cat/dog sensitization^‡^	146/215 (67.9)	137/175 (78.3)	**0.030**
**Peanut sensitization^§^**			
**SPT peanut extract**			
Positive	176/212 (83.0)	144/175 (82.3)	0.956
Allergen/histamine wheal ratio, *median (IQR)*	0.78 (0.57–1.00)	1.07 (0.64–1.80)	<0.001^*^
**ImmunoCAP peanut extract**			
Positive	144/209 (68.9)	140/167 (83.8)	0.001^*^
IgE level, *median (IQR)*	0.95 (0.22–3.23)	2.21 (0.75–12.84)	<0.001^*^
Microarray peanut allergens^**^			
**Ara h 1**			
Positive	26/176 (14.8)	54/144 (37.5)	<0.001^*^
IgE level, *median (IQR)*	0.00 (0.00–0.00)	0.00 (0.00–0.83)	**0.004**
**Ara h 2/6**			
Positive	19/176 (10.8)	56/144 (38.9)	<0.001^*^
IgE level, *median (IQR)*	0.00 (0.00–0.00)	0.00 (0.00–6.89)	<0.001^*^
**Ara h 3/3.02**			
Positive	10/176 (5.7)	43/144 (29.9)	<0.001^*^
IgE level, *median (IQR)*	0.00 (0.00–0.00)	0.00 (0.00–0.49)	**0.001**
**Ara h 8**			
Positive	112/176 (63.6)	67/144 (46.5)	**0.003**
IgE level, *median (IQR)*	0.44 (0.00–1.21)	0.12 (0.00–0.82)	0.096

*All measurements are in n/N (%) unless otherwise specified. P-values indicate difference between patients with mild-to-moderate and patients with severe allergic symptoms to peanut. Bold indicates p < 0.05*.

* *Differences remained significant after Bonferroni correction*.

‡ *Reported symptoms + matching sensitization by SPT or ImmunoCAP*.

§ *Not all patients had complete testing for peanut sensitization*.

** *Allergen components measured by microarray in 322 patients. IQR, interquartile range; SPT, skin prick test*.

### Demographic and Clinical Characteristics Associated With Severity of Probable PA

**Figure 1:**
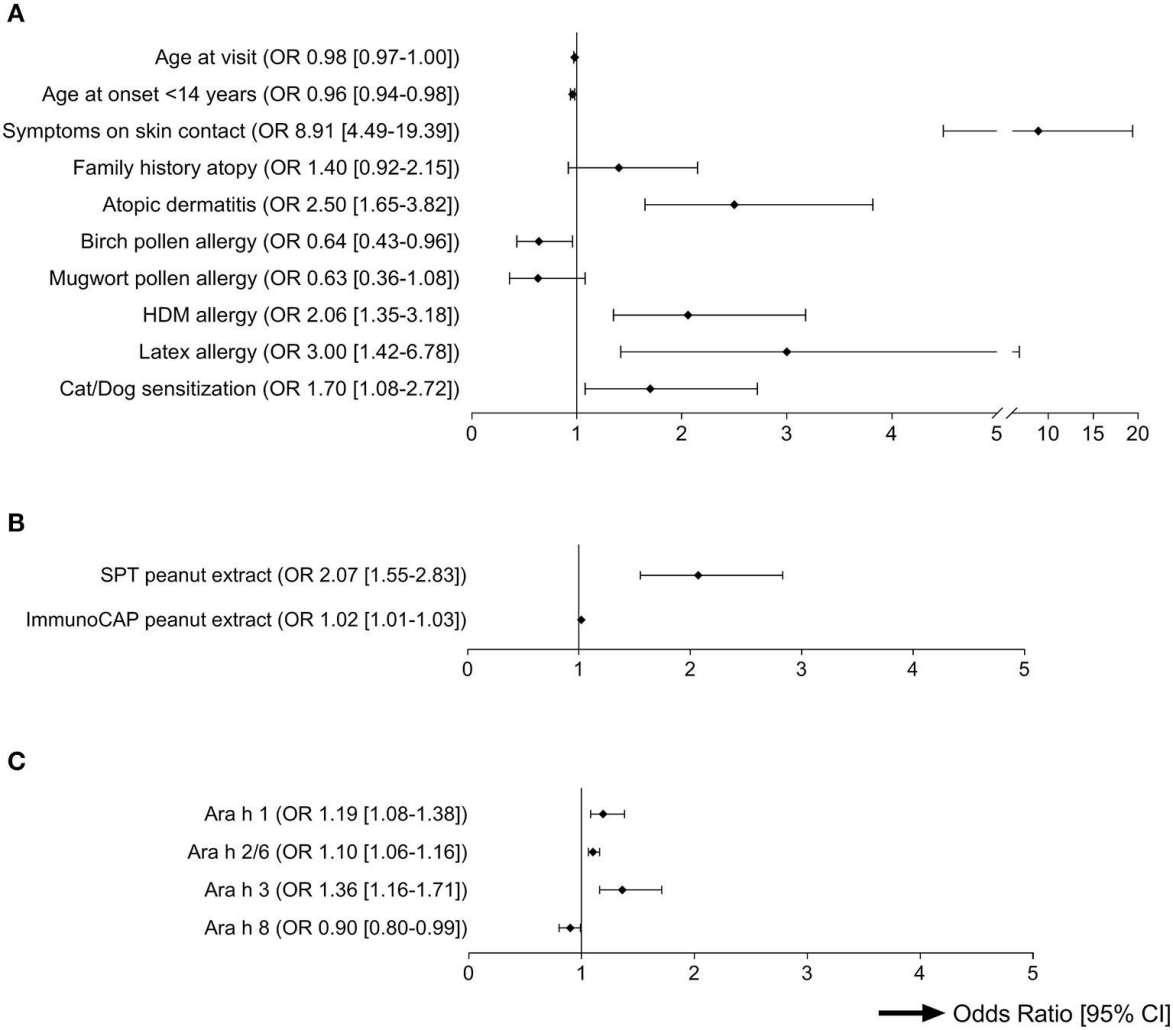
Univariable Odds Ratios for prediction of severity of probable PA (*p* < 0.2). This forest plot shows the ORs and their respective confidence intervals from univariable analyses of all predictors for severity of probable peanut allergy with *p* < 0.2 (Table 1). All variables under **(B)** and **(C)**, and in **(A)** “age at visit” were entered as continuous variables. All other variables were dichotomous. **(A)** Demographics and clinical background. **(B)** Sensitization to peanut extract. **(C)** Sensitization to peanut components.

Frequencies of demographic and clinical background characteristics of patients with mild-to-moderate and those with severe probable PA are presented in Table 1 and Figure 1. Subjects with the severe phenotype were younger than those with the mild-to-moderate phenotype, and manifestation of probable PA more often occurred before the age of 14 years. Subjects with the severe phenotype were more likely to have symptoms elicited by skin contact with peanut, AD, HDM allergy, latex allergy or sensitization to cats and/or dogs, but less likely to be allergic to birch pollen.

### Measures of IgE Sensitization Associated With Severity of Probable PA

Of subjects with probable PA, 320/387 (83%) had a positive SPT and 284/376 (76%) had a positive ImmunoCAP test to peanut extract (Table 1), and 240/370 (65%) tested positive to both tests. The allergen/histamine wheal ratios and levels of IgE to peanut extract were significantly higher in patients with severe symptoms than in patients with mild-to-moderate symptoms (Table 1 and Figure 1).

Microarray was performed in 322 of 391 (82%) subjects with probable PA, and 230/322 (71%) were sensitized to at least one peanut component. All 27 component-sensitized subjects who were not sensitized to peanut extract in SPT or ImmunoCAP, were sensitized to Ara h 8 (Supplementary Table 2). Overall, sensitization to Ara h 8 was most common, and associated with mild-to-moderate probable PA (although not significantly after Bonferroni correction). Sensitization to Ara h 1, Ara h 2/6 or Ara h 3 was associated with severe probable PA, and IgE levels to these components were significantly higher in those with severe symptoms (Table 1 and Figure 1). Of the 179 subjects with IgE to Ara h 8 (Table 1), 48 (27%) also tested positive to Ara h 1, Ara h 2/6 or Ara h 3. Co-sensitization to storage proteins in those sensitized to Ara h 8 was associated with a more severe phenotype (*p* = 0.009).

Regarding foods other than peanut, IgE levels to extract from other legumes, soybean and lentil, were higher in subjects with severe probable PA than in those with mild-to-moderate probable PA (Supplementary Table 3). At a molecular level, subjects with severe probable PA were significantly more often sensitized to soybean Gly m 5 (7S globulin) and Gly m 6 (11S globulin), hazelnut Cor a 11 (7S globulin), walnut Jug r 2 (7S globulin), and sesame Ses i 1 (2S albumin) (Supplementary Table 4). IgE levels to peach, apple and celery extract were higher in subjects with mild-to-moderate probable PA than in subjects with severe probable PA. The mild-to-moderately peanut allergic subjects were more often sensitized to PR10 proteins Gly m 4 (soybean), Cor a 1 (hazelnut), and Mal d 1 (apple).

### Discriminating Between Mild-To-Moderate and Severe Probable PA

The AUCs of single tests (SPT peanut extract, ImmunoCAP peanut extract, microarray peanut components) for discriminating between patients with mild-to-moderate and severe probable PA ranged from 0.54 to 0.64 (Supplementary Table 5). The accuracy of SPT wheal ratio and of peanut extract and component IgE levels at specific cutpoints, are shown in Supplementary Table 6. The most discriminative model combining microarray results comprised IgE levels to Ara h 2/6 and Ara h 8, with an AUC of 0.65 (95% CI 0.63–0.66). The AUCs of our three models taking demographic and clinical factors as starting point, and combining those with markers for peanut extract and component sensitization, were significantly larger than the AUCs of the single peanut sensitization tests (P_De Long′s test_ < 0.001) (Table 2 and Supplementary Table 5).

**Table 2:** Prediction models for severity of probable PA.

	**Model I:** **Demographics & clinical background**		**Model II:** **Model I +** **sensitization to peanut extract**		**Model III:** **Model II +** **sensitization to peanut components**	
	**OR**	**95%-CI**	**OR**	**95%-CI**	**OR**	**95%-CI**
Age at onset <14 years	1.34	0.84–2.13	1.16	0.77–1.77	1.15	0.77–1.70
Female sex	1.27	0.82–1.97	1.30	0.83–2.04	1.29	0.84–1.99
Family atopy	1.35	0.85–2.15	1.35	0.85–2.16	1.31	0.85–2.01
Atopic dermatitis	1.51	0.93–2.44	1.43	0.90–2.27	1.46	0.91–2.35
Symptoms skin contact	5.71	2.98–10.93	4.78	2.47–9.25	4.57	2.33–8.89
Birch pollen allergy	0.61	0.37–1.01	0.63	0.38–1.04	0.57	0.44–1.15
HDM allergy	1.58	0.98–2.56	1.47	0.91–2.36	1.43	0.91–2.25
Latex allergy	1.71	0.73–4.00	1.73	0.78–3.86	1.67	0.74–1.58
SPT peanut extract			1.26	0.98–1.61	1.22	0.94–1.58
IgE level peanut extract			1.01	1.00–1.01	1.00	1.00–1.01
IgE level Ara h 1					1.02	0.95–1.05
IgE level Ara h 2/6					1.01	0.98–1.04
IgE level Ara h 8					0.95	0.87–1.03
Intercept	−1.25		−1.40		−1.36	
**AUC (95%-CI)**	0.74 (0.72–0.75)		0.74 (0.73–0.76)		0.75 (0.74–0.77)	

*The area under the curve (AUC) indicates the ability of the model to discriminate between patients with mild-to-moderate and patients with severe allergic symptoms to peanuts. HDM, house dust mite; SPT, skin prick test*.

In the first model, female sex, age at onset of PA < 14 years, symptoms elicited by skin contact with peanut, family atopy, AD, birch pollen allergy, HDM allergy, and latex allergy, were selected by Lasso regression. All determinants, except for birch pollen allergy, were associated with severe probable PA. This combination of clinical and demographic factors resulted in an AUC of 0.74 (95% CI 0.72–0.75). Lasso regression selected SPT wheal size ratio and ImmunoCAP IgE level to peanut extract (both associated with severe PA) as additionally contributing variables in model II, and IgE to Ara h 1 and Ara h 2/6 (severe) and Ara h 8 (mild-to-moderate) in model III, although AUC showed only a limited increase (Table 2). After model III, no IgE levels to foods and food components other than peanut were additionally selected to help discriminate between mild-to-moderate and severe PA.

### Discriminating Between Mild-To-Moderate and Severe Symptoms to Peanut in Subjects Who Underwent DBPCFC, or Experienced Severe Life-Threatening Anaphylaxis

Overall, 52/393 subjects with probable PA agreed to undergo DBPCFC, of which 4 were excluded from analyses because of incomplete data. A total of 91 subjects were included in the subgroup analysis: 47 subjects with no or mild-to-moderate symptoms during DBPCFC (18 subjects with no symptoms, 22 with mild-to-moderate symptoms, 7 placebo-reactors), and 44 subjects with severe symptoms during DBPCFC (*N* = 1) or a convincing history of severe life-threatening anaphylaxis, leading to exclusion from DBPCFC (*N* = 43). Details on demographics, clinical variables, SPT and IgE results are available from Supplementary Table 7.

Just like for probable PA, symptoms elicited by skin contact with peanut (associated with severe PA), female sex (severe), family atopy (severe), birch pollen allergy (mild-to-moderate) and HDM allergy (severe) were selected as demographic and clinical predictors for PA in the DBPCFC/anaphylaxis subgroup, with additionally lower age at visit (mild-to-moderate) and grass pollen allergy (mild-to-moderate). IgE to peanut extract (severe) was selected in model II, but no longer in model III, where IgE to Ara h 1 (severe) and Ara h 8 (severe) were favored. The AUC of these models ranged from 0.68 to 0.72 for discriminating between mild-to-moderate and severe PA as determined in the DBPCFC/anaphylaxis subgroup, and did not differ significantly from the AUCs of individual extract- and allergen-based tests (Table 3 and Supplementary Table 5).

**Table 3:** Prediction models for severity of PA according to DBPCFC or history of anaphylaxis.

	**Model I:** **Demographics & clinical background**		**Model II:** **Model I +** **sensitization to peanut extract**		**Model III:** **Model II +** **sensitization to peanut components**	
	**OR**	**95%-CI**	**OR**	**95%-CI**	**OR**	**95%-CI**
Age at visit	0.95	0.90–1.01	0.96	0.91–1.02	0.96	0.90–1.03
Female sex	2.37	0.69–8.14	2.43	0.62–9.57	2.64	0.34–20.77
Family atopy	5.53	1.45–21.06	4.97	1.27–19.45	5.16	1.15–23.14
Symptoms skin contact	9.93	2.22–44.39	9.00	1.83–44.33	8.69	0.97–77.97
Birch pollen allergy	0.64	0.19–2.14	0.61	0.18–2.14	0.57	0.12–2.65
Grass pollen allergy	0.39	0.09–1.63	0.40	0.09–1.76	0.43	0.08–2.28
HDM allergy	3.11	0.75–12.84	2.96	0.67–12.99	2.85	0.64–12.59
IgE level peanut extract			1.01	0.99–1.03		
IgE level Ara h 1					1.08	0.71–1.63
IgE level Ara h 8					1.06	0.75–1.48
Intercept	−1.33		−1.60		−1.74	
**AUC (95%-CI)**	0.68 (0.65–0.72)		0.72 (0.68–0.75)		0.71 (0.67–0.74)	

*The area under the curve (AUC) indicates the ability of the model to discriminate between patients with mild-to-moderate and patients with severe allergic symptoms to peanuts. HDM, house dust mite; SPT, skin prick test*.

## Discussion

The current study provides insight into the clinical profiles of subjects with mild-to-moderate and severe probable PA, and quantifies the relative importance of information obtained during diagnostic work-up of PA for prediction of severity. Sex, age at onset of PA, symptoms elicited by skin contact with peanut, family atopy, AD (ever), birch pollen allergy, HDM allergy, latex allergy, peanut extract SPT wheal ratio, and IgE levels to peanut extract, Ara h 1, 2/6 and 8, were found to be independently associated with severity, of which only birch pollen allergy and IgE to Ara h 8 were associated with a mild-to-moderate phenotype. A model combining these determinants led to optimal discrimination between mild-to-moderate and severe probable PA (cross-validated AUC 0.75), but measures of peanut sensitization contributed only limited predictive value in addition to clinical background determinants alone.

It was intriguing that some of the strongest independent predictors from clinical background associated with severe probable PA were skin-related: having symptoms elicited by skin contact with peanut, AD (ever), or latex allergy (Figure 1). Exposure to food allergens in early life via the skin has been proposed to play an important role in allergic sensitization (31). Loss-of-function mutations in genes encoding the skin component filaggrin are related to a disrupted skin barrier, are often seen in children with AD, and are associated with IgE sensitization and allergy to foods in general (32, 33), and peanut specifically (33–36). Little has been reported on the relationship between AD and severity of food allergic reactions, but in agreement with our findings, Van der Leek et al. also found that peanut allergic children reporting skin contact reactions to peanut were more likely to experience severe peanut allergic reactions (37). Similarly, our prediction models developed for hazelnut and walnut allergy also contained AD (hazelnut and walnut), latex allergy (hazelnut), and symptoms elicited by skin contact (walnut) as predictors for severe reactivity (20, 21). Altogether, cutaneous sensitivity may be a marker for severe food allergy.

The only independent determinants to be associated with mild-to-moderate probable PA, were birch pollen allergy and sensitization to Ara h 8, a PR-10 protein homologous to major birch pollen allergen Bet v 1. Birch pollen-related FA is one of the most common types of plant source FA in adults in (especially Northern and Central) Europe and generally presents with mild (often isolated oral allergy) symptoms (1, 38). The frequent occurrence of this condition is reflected in our study population−41% of subjects with mild-to-moderate PA had isolated OAS, of which 73% were sensitized to Ara h 8, making birch pollen-related PA plausible.

Interestingly, all subjects with probable PA who were not sensitized to peanut extract in SPT or ImmunoCAP, were found to be sensitized to Ara h 8 (Supplementary Table 2). The peanut PR-10 protein is apparently underrepresented in peanut extract. This suggests that subjects with birch pollen related PA are not well-detected with peanut extract, which partly explains why SPT wheal size and IgE level to peanut extract are associated with severe probable PA. Our findings were similar for walnut allergy, where the majority of subjects with negative extract-based tests were sensitized to walnut PR-10 protein Jug r 5 (21). In contrast, sensitization to hazelnut extract, which is spiked with hazelnut PR-10 protein Cor a 1, is more common in subjects with mild-to-moderate hazelnut allergy (20). In the awareness that the association between extract-based testing and severity of PA was limited, these observations still underline the importance of understanding the allergen composition of food extracts for clinical interpretation of extract-based test results.

Our data showed that levels of IgE to peanut storage proteins Ara h 1, 2/6 and 3 (and also to other legumes,' tree nuts' and seeds' storage proteins) were significantly higher in subjects with severe probable PA, in accordance with several previous studies in primarily adult populations (7, 16, 39, 40). Of the individual tests for IgE sensitization to peanut extract or components, IgE to Ara h 2/6 had the strongest ability to discriminate between mild-to-moderate and severe probable PA, but the AUC only reached 0.64 (Supplementary Table 5). This observation indicated that, although IgE levels to Ara h 1, 2/6 and 3 correlated significantly with severity, they could not be used independently to predict severity of probable PA in an individual patient. These findings were in support of those previously reported by Klemans et al. who also found that IgE to Ara h 2 was associated with severity of PA in their adult population, but could not discriminate well between mild and severe PA in individual patients, with comparable AUCs of 0.58 for severity based on patient history and 0.65 for severity based on DBPCFC (7).

In the current study, IgE to peanut extract (in both SPT and ImmunoCAP) and to peanut storage proteins Ara h 1 and Ara h2/6, were found to contribute to an increased risk of severe probable PA in multivariable analyses. However, the negligible increase of the AUC after addition of measures of peanut IgE sensitization (in model II and III) to information from clinical background (model I), implies that clinical background is most useful for predicting severity of probable PA in an individual patient, and patient history can detect most of the variation explained by differences in IgE levels. To our knowledge, only one previous study, by Petterson et al. assessed prediction of *severity* of PA using a combination of variables from clinical background and measures of IgE sensitization (only peanut extract), but in a pediatric population and using linear regression (22). They conclude that reaction severity is largely unpredictable, but the differences in methodological approach prevent in-depth comparison to our study results. Some studies suggest that other laboratory predictors than taken into account in our study may also contribute to prediction of severe PA, such as epitope diversity (combined rather than isolated recognition of Ara h 1, 2 and 3) (41, 42), sIgE/sIgG_4_ ratios (15, 43), or results from the basophil activation test (BAT) (15, 44). Especially the BAT has recently been explored independently and as part of multivariable approaches for prediction of PA severity in several studies. The promising results, albeit in primarily pediatric populations, suggest that the BAT may have the potential to truly enhance prediction of PA severity in the coming years (43, 45–48).

Other recommendations for improving prediction of severity of PA in future research, building on the findings in the current study, would be to use ImmunoCAP rather than the less sensitive microarray for measurement of component-specific IgE, and to include other potentially relevant peanut components, like profilin Ara h 5, 2S albumin Ara h 7 and lipid transfer protein (LTP) Ara h 9 (16, 19, 49, 50). The latter is a major peanut allergen in Southern Europe and may contribute to higher predictive accuracy in those regions (16, 51). In our population, ~16% of subjects with probable PA were sensitized to peach LTP Pru p 3 (Supplementary Table 4), which is generally considered the primary source of LTP sensitization (52–54). A previous EuroPrevall study revealed that 73% of peanut allergic subjects with Pru p 3 sensitization were sensitized to Ara h 9 (16), which suggests that up to 12% of the subjects with probable PA in our population may have Ara h 9 sensitization. That said, it remains unclear whether knowledge of Ara h 9 sensitization would contribute to prediction of PA severity, as LTP sensitization has been linked to both mild and severe food allergy phenotypes (52), and was not associated with systemic reactions to peanut by Ballmer-Weber et al. (16). In accordance, we also found that sensitization to Pru p 3 was not significantly associated with mild-to-moderate or severe PA in our population (Supplementary Table 4), nor did IgE levels to Pru p 3 improve prediction of PA severity in the multivariable model. The results from the current studies are, for the largest part, based on subjects from birch-endemic areas. It is important to realize that we made the conscious decision to include subjects with likely birch-pollen related PA in our population, even though pollen-related food allergy is considered a separate clinical entity by some. Exclusion of these patients would make the clinical relevance of our findings much more limited for the average presenting outpatient population in most countries in this study. In future research, further specification of the study population to only include subjects from regions with similar pollen exposure, or only children or adults, could further refine prediction and clinical applicability of findings.

One might consider the main limitation of our study that the primary outcome measure was based on self-reported symptoms rather than symptoms during challenge testing. For this reason, we made sure only subjects with IgE sensitization to peanut extract or components were included, and additionally explored the results of our analyses in the subgroup of subjects who underwent challenge testing or were excluded from challenge testing because of a history of severe anaphylaxis. We found it reassuring that there was considerable overlap in independent predictors. It was surprising that Ara h 8 tended to be associated with a more severe phenotype of PA in the DBPCFC/anaphylaxis group, for which we have no clear explanation other than that the subgroup likely does not accurately represent an unselected population of subjects with PA, as subjects with no reaction or reaction to placebo were included in the sub-analysis and the classification of severity in subjects with life-threatening anaphylaxis was based on self-reported symptoms. It is therefore important to realize that the sub-analysis was merely explorative. We also point out that reaction severity based on self-reported symptoms may better reflect real life than reaction severity estimated by challenge, because of exclusion and stopping criteria, and the disinclination of patients who experience severe reactions to undergo or complete a burdensome challenge. As a result of the latter, dietary avoidance advice and medical prescriptions in daily practice are often decided based on clinical history and measurements of IgE sensitization, making models predicting severity of probable PA particularly interesting. We used penalized regression to prevent overfitting of our models to the population in which they were developed, but as with all prediction models, the models should still be validated in an external population.

To our knowledge, this is the first study to evaluate the individual and combined contribution of clinical background, extract-based tests, and CRD, for prediction of PA severity in a primarily adult population. The penalized regression method increases the generalizability of results, and the standardized approach facilitates comparison to similar models designed for tree nuts. Although not superimposable, clinical profiles for hazelnut and walnut displayed clear similarities. However, it was interesting to observe that measurements of IgE sensitization only contributed minimally to prediction of severity of probable PA, in contrast to the models for severity of hazelnut or walnut allergy. Clinical background determinants were clearly most valuable for predicting severity of probable PA in an individual patient. It will be interesting to validate and further expand these models in other populations to increase predictive accuracy, and to develop models according to the same approach in other food groups for comparative purposes.

## Data Availability Statement

The datasets presented in this article are not readily available because the EuroPrevall datasets are only available by request to the original coordinator of the EuroPrevall Project, E. N. Clare Mills. A Data Access Committee decides whether the data will be made accessible. Requests to access the datasets should be directed to clare.mills@manchester.ac.uk.

## Ethics Statement

The studies involving human participants were reviewed and approved by the local ethical committee of each of the 12 participating centers. Written informed consent to participate in this study was provided by the participants' legal guardian/next of kin.

## Author Contributions

EM and RR: coordination of EuroPrevall project. MF-R: coordinator of EuroPrevall outpatient clinic survey design of survey and protocol writing. MF-R and BB-W: training of the clinical partners and anaphylaxis board. BB-W: coordination of the food challenges in EuroPrevall. MF-R, BB-W, AK, RA, SB, FB, MC, RD, PF, DG, MJ-C, MK, TK, TP, AP, IR, SS, T-ML, and EV: recruitment and clinical investigation of the patients and food challenge performance. KH-S: coordination of allergen bank. JL: generation of reagents and experimental IgE assays. SV, SAV, and LJ: immunoCAP IgE testing (extract based). MF-R, LB, and CF-P: database design, data collection, and data cleaning. LB, CF-P, NP, and MF-R: monitoring data collection and data cleaning. MD and SL: statistical data analyses and writing of the manuscript. AZ and PW: supervision of the statistical data analyses. MF-R, BB-W, AK, RA, LB, SB, FB, MC, RD, CF-P, PF, DG, KH-S, MJ-C, LJ, MK, TK, JL, NGP, TP, NP, AP, IR, SS, AS, EV, SAV, SV, PW, EM, T-ML, AZ, and RR: critical review of the manuscript. All authors contributed to the article and approved the submitted version.

## Conflict of Interest

Outside of submitted work: MF-R reported grants and personal fees from Aimmune Therapeutics and Diater, personal fees from DBV, Allergy Therapeutics, GSK, HAL Allergy, Novartis, ThermoFisher Scientific, and SPRIM. BB-W reported personal fees from ThermoFisher Scientific. FB reported personal fees from Aimmune; grants from Stallergènes Greer, Chiesi, Mundipharma, Novartis, and Regeneron; and board membership for DVB, Stallergènes Greer, Novartis, ALK, Mundipharma, Boehringer, AstraZeneca, Medapharma, and Boston Scientific. JL was an employee of ThermoFisher Scientific. NP reported personal fees from Novartis, Nutricia, HAL Allergy BV, Menarine/Faes Farma, Sanofi, Mylan/Meda, Biomay, AstraZeneca, GSK, MSD, ASIT Biotech, Boehringer Ingelheim; and grants from Gerolymatos International SA, and Capricare. SV reported personal fees from Ärzteverband Deutscher Allergologen, Swiss Society for Allergy and Immunology, Schattauer Allergologie Handbuch, Elsevier Nahrungsmittelallergien und Intoleranzen, Karger Food Allergy: Molecular Basis and Clinical Practice, and Pharmacon. EM reported grants from Reacta Biotech; and was shareholder of Reacta Biotech Ltd. RR reported personal fees from HAL Allergy BV, Citeq BV, Angany Inc., and ThermoFisher Scientific. The remaining authors declare that the research was conducted in the absence of any commercial or financial relationships that could be construed as a potential conflict of interest.

## Abbreviations

SPT: Skin prick test
AD: Atopic Dermatitis
CRD: Component-resolved diagnosis
DBPCFC: Double-blind placebo-controlled food challenge
LTP: Lipid transfer protein
OAS: Oral allergy syndrome
OR: Odds Ratio.
