# Supplementary material for: Estimating the Risk of Severe Peanut Allergy Using Clinical Background and IgE Sensitization Profiles
Source: Front Allergy. 2021 Jun 7;2:670789. doi: 10.3389/falgy.2021.670789 (PMC8974676; doi:10.3389/falgy.2021.670789)
Supplement: Supplementary file 1 [file Data_Sheet_1.pdf]

Supplementary Files

Table S1. Missing data in variables included for Lasso regression

Variable	Number of missings
Age at visit	0
Sex	0
Age at onset of symptoms peanut allergy	8
Symptoms upon skin contact peanut	55
Family history of atopic disease	7
Atopic dermatitis (ever)	6
Birch pollen allergy (reported)	8
IgE birch extract	20
SPT birch extract	22
Grass pollen allergy (reported)	8
IgE grass extract	20
SPT grass extract	22
Mugwort pollen allergy (reported)	8
IgE mugwort extract	20
SPT mugwort extract	25
House dust mite allergy (reported)	32
IgE house dust mite extract	20
SPT house dust mite extract	21
Latex allergy (reported)	33
IgE latex	42
IgE cat	20
IgE dog	42
SPT peanut extract	6
IgE peanut extract (ImmunoCAP)	17
Ara h 1 (microarray)	73
Ara h 2/6 (microarray)	73
Ara h 3/3.02 (microarray)	73
Ara h 8 (microarray)	73

Total N = 393. Values for these missing data were estimated using multiple imputation procedures, for which all of the above determinants were included as covariates, along with reported symptoms (0 missings), centre, and reported allergy, SPT, ImmunoCAP and microarray results for foods other than peanut. *SPT, skin prick test.*

Table S2. IgE to peanut components in subjects with negative peanut SPT and ImmunoCAP

	**Negative SPT and ImmunoCAP peanut extract (N=27)**	
	**N microarray positive***	**IgE level**, *median (IQR)*
Ara h 1	2/27	0.30; 0.31
Ara h 2/6	0/27	NA
Ara h 3	0/27	NA
Ara h 8	27/27	0.51 (0.68-4.10)

* *IgE ≥0.3 ISU/L. NA, not applicable*, because 0 subjects sensitized. Of the 27 patients, 10 (37%) had isolated oral allergy symptoms, 11 (41%) skin symptoms, 5 (19%) eye symptoms, 7 (26%) upper airway symptoms, 4 (15%) gastrointestinal symptoms, 5 (19%) lower airway symptoms, 1 (4%) cardiovascular symptoms and 1 (4%) neurological symptoms. Overall, 7/27 (26%) had severe probable PA.

Table S3. Sensitization to food extracts other than peanut in subjects with mild-to-moderate and severe probable PA

Food extract	Measurement	Mild-to-moderate (N=216)		Severe (N=177)		p
Soybean	n positive/N total, %	94/209	45.0	83/167	49.7	0.419
	IgE level median, IQR	0.25	0.05-0.79	0.35	0.07-1.54	**0.022**
Lentil	n positive/N total, %	89/208	42.8	83/168	49.4	0.240
	IgE level median, IQR	0.24	0.05-0.90	0.33	0.06-1.95	**0.020**
Hazelnut	n positive/N total, %	168/208	80.8	135/168	80.4	1.000
	IgE level median, IQR	4.74	0.73-24.89	3.55	0.64-19.53	0.065
Walnut	n positive/N total, %	91/208	43.8	76/168	45.2	0.854
	IgE level median, IQR	0.25	0.04-1.36	0.27	0.06-1.09	0.512
Sesame seed	n positive/N total, %	111/208	53.4	106/167	63.5	0.062
	IgE level median, IQR	0.40	0.15-1.40	0.71	0.21-2.08	0.461
Peach	n positive/N total, %	168/207	81.2	107/168	63.7	**<0.001***
	IgE level median, IQR	2.59	0.59-7.20	1.24	0.18-4.35	**0.006**
Apple	n positive/N total, %	147/207	71.0	104/168	61.9	0.079
	IgE level median, IQR	1.39	0.27-4.97	0.77	0.16-2.86	**0.038**
Kiwi	n positive/N total, %	109/207	52.7	95/168	56.5	0.517
	IgE level median, IQR	0.40	0.10-1.68	0.42	0.08-1.27	0.074
Tomato	n positive/N total, %	111/208	53.4	87/168	51.8	0.841
	IgE level median, IQR	0.41	0.10-1.91	0.38	0.09-1.52	0.128
Carrot	n positive/N total, %	121/208	58.2	95/168	56.5	0.832
	IgE level median, IQR	0.51	0.11-2.54	0.55	0.08-1.76	0.064
Celery	n positive/N total, %	122/208	58.7	97/168	57.7	0.941
	IgE level median, IQR	0.68	0.14-2.73	0.54	0.08-1.98	**0.043**

Sensitization was considered positive at IgE levels ≥0.35 kU/L. The p-value indicates the difference between mild-to-moderate and severe probable peanut allergy subjects. **Bold** indicates p<0.05. *Differences remained significant after Bonferroni correction.

Table S4. Sensitization to food allergens other than peanut allergens in subjects with mild-to-moderate and severe probable PA

Food source	Allergen	Mild-to-moderate (N=216)		Severe (N=177)		p
		**n positive/N total**	**%**	**n positive/N total**	**%**	
Soybean	Gly m 4	95/176	54.0	45/144	31.2	**<0.001***
	Gly m 5	7/176	4.0	24/144	16.7	**<0.001***
	Gly m 6	6/176	3.4	25/144	17.4	**<0.001***
Hazelnut	Cor a 1.0401	115/176	65.3	71/144	49.3	**0.005**
	Cor a 2	32/176	18.2	17/144	11.8	0.156
	Cor a 8	26/176	14.8	23/144	16.0	0.888
	Cor a 9	30/176	17.0	23/144	16.0	0.916
	Cor a 11	2/176	1.1	17/144	11.8	**<0.001***
Walnut	Jug r 2	10/176	5.7	20/144	13.9	**0.021**
	Jug r 4	2/176	1.1	7/144	4.9	0.084
Sesame seed	Ses i 1	0/176	0.0	5/144	3.5	**0.018**
	Ses i 2	1/176	0.005	5/144	3.5	0.094
	Ses i 3	35/176	19.9	24/144	16.7	0.553
Peach	Pru p 1	108/176	61.4	74/144	51.4	0.093
	Pru p 3	34/176	19.3	16/144	11.1	0.063
Apple	Mal d 1	82/176	46.6	49/144	34.0	**0.031**
	Mald 2	2/176	1.1	2/144	1.4	1.000
	Mal d 3	30/176	17.0	21/144	14.6	0.656
	Mal d 4	34/176	19.3	29/144	20.1	0.966
Kiwi	Act d 1	4/176	2.3	10/144	6.9	0.054
Tomato	Lyc e 3	22/176	12.5	15/144	10.4	0.686
Carrot	Dau c 1.0201	24/176	13.6	24/144	16.7	0.550
	Dau c 1.0103	9/176	5.1	8/144	5.6	1.000
	Dauc 4	38/176	21.6	32/144	22.2	1.000
Celery	Api g 1.01	36/176	20.5	24/144	16.7	0.472
	Api g 4	46/176	26.1	39/144	27.1	0.949
	Api g 5	37/176	21.0	26/144	18.1	0.601

Sensitization was considered positive at IgE levels ≥0.3 ISU/L. The p-value indicates the difference between mild-to-moderate and severe probable PA subjects. **Bold** indicates $p<0.05$. *Differences remained significant after Bonferroni correction.

Table S5. Area under the ROC-curve of individual and combined tests for prediction of severity of PA

**Test**	**Probable peanut allergy**		**DBPCFC/anaphylaxis**	
	**AUC**	**95%-CI**	**AUC**	**95%-CI**
**Peanut extract**				
SPT	0.63	0.61-0.65	0.63	0.60-0.67
ImmunoCAP	0.63	0.62-0.65	0.72	0.69-0.75
**Peanut allergens (microarray)**				
Ara h 1	0.62	0.59-0.64	0.70	0.66-0.75
Ara h 2/6	0.64	0.61-0.66	0.70	0.60-0.81
Ara h 3/3.02	0.60	0.58-0.63	0.69	0.64-0.73
Ara h 8	0.54	0.50-0.61	0.47	0.43-0.51
**CRD only***				
Ara h 2/6 & Ara h 8*	0.65	0.63-0.66	-	-
Ara h 1 & Ara h 2/6*	-	-	0.70	0.66-0.75
**Models****				
Model I	0.74†	0.72-0.75†	0.68	0.65-0.72
Model II	0.74†	0.73-0.76†	0.72	0.68-0.75
Model III	0.75†	0.74-0.77†	0.71	0.67-0.74

The areas under the curve (AUC) and the 95% confidence intervals (95%-CI) indicate the ability to discriminate between patients with mild-to-moderate and patients with severe allergic symptoms to peanuts. AUCs for SPT, peanut extract and allergen components by microarray were averaged over the 10 imputed datasets. *Allergens selected by Lasso regression when combining peanut allergens measured by microarray. For probable peanut allergy, the model included Ara h 2/6 and Ara h 8. For the DBPCFC group, the model included Ara h 1 and Ara h 2/6. **As shown in table 3. †Significantly larger ($p<0.001$) than the AUC of individual extract-based and allergen-based tests (De Long's test). *CI, confidence interval; DBPCFC, double-blind placebo-controlled food challenge; SPT, skin prick test.*

Table S6. Accuracy of individual diagnostic tests and models for severity of peanut allergy

Individual test	Positivity threshold		Mild-to-moderate	Severe	Sensitivity	95%-CI	Specificity	95%-CI	PPV	95%-CI	NPV	95%-CI
**SPT**	**0.50**	≥	173	143	81.7	75.2-87.1	18.4	13.4-24.3	45.2	39.7-50.9	54.9	42.7-66.8
**peanut extract**		<	39	32								
	**0.29**	≥	181	150	**85.1**	**79.6-90.5**	14.6	10.2-20.1	45.3	39.8-50.8	55.4	41.5-68.7
		<	31	25								
	**2**	≤	11	34	19.4	13.8-26.1	**94.8**	**90.9-97.4**	75.6	60.5-87.1	58.8	53.4-64.0
		>	201	141								
**ImmunoCAP peanut**	**0.35**	≥	144	140	83.8	77.4-89.1	31.1	24.9-37.9	49.3	43.3-55.3	70.7	60.2-79.7
**extract**		<	65	27								
	**0.007**	≥	182	158	**94.6**	**90.0-97.5**	12.9	8.7-18.2	46.5	41.1-51.9	75	57.8-87.9
		<	27	9								
	**25.7**	≥	10	33	19.8	14.0-26.6	**95.2**	**91.4-97.7**	76.7	61.4-88.2	59.8	54.3-65.1
		<	199	134								
**Microarray**	**0.30**	≥	26	54	37.5	29.6-46.0	87.5	81.7-92.0	71.0	59.5-80.9	63.1	56.7-69.2
**Ara h 1**		<	150	90								
	**0.10**	≥	34	63	**43.8**	**35.5-52.3**	80.7	74.1-86.2	65.0	54.6-74.4	63.7	5.0-70.0
		<	142	81								
	**0.85**	≥	8	36	25.0	18.2-32.9	**95.5**	**91.3-98.0**	81.8	67.3-91.8	**60.9**	**54.8-66.7**
		<	168	108								
**Microarray**	**0.30**	≥	19	56	38.9	30.9-47.4	89.2	83.7-93.4	74.7	63.3-84.0	64.1	57.7-70.1
**Ara h 2/6**		<	157	88								
	**0.11**	≥	19	59	**41.0**	**32.9-49.8**	89.2	83.7-93.4	75.6	64.6-84.7	64.9	58.5-70.9
		<	157	85								
	**8.27**	≥	8	34	23.6	16.9-31.4	**95.5**	**91.2-98.0**	81.0	65.9-91.4	60.4	54.4-66.2
		<	168	110								
**Microarray**	**0.30**	≥	10	43	29.9	22.5-38.0	94.3	89.8-97.2	81.1	68.0-90.6	62.2	56.1-68.0
**Ara h 3**		<	166	101								
	**0.19**	≥	12	47	**32.6**	**25.1-40.9**	93.2	88.4-96.4	79.7	67.2-89.0	62.8	56.7-68.7
		<	164	97								
	**0.26**	≥	8	33	22.9	16.3-30.6	**95.5**	**91.2-98.0**	80.5	65.1-91.2	60.2	54.2-66.0
		<	168	111								
**Microarray**	**0.30**	≥	112	67	46.5	38.2-55.0	36.4	29.3-43.9	37.4	30.3-45.0	45.4	37.0-54.0
**Ara h 8**		<	64	77								
	**0.10**	≥	119	86	**59.7**	**51.2-67.8**	32.4	25.5-39.8	42.0	35.1-49.0	49.6	40.1-59.0
		<	57	58								
	**8.53**	≥	8	1	0.7	0.3-8.0	**95.5**	**91.2-98.0**	11.1	0.3-48.2	54.0	48.3-59.7
		<	168	143								

Table S6. Accuracy of individual diagnostic tests and models for severity of peanut allergy (continued)

**Model**	**Positivity threshold**		**Mild-to-moderate**	**Severe**	**Sensitivity**	**95%-CI**	**Specificity**	**95%-CI**	**PPV**	**95%-CI**	**NPV**	**95%-CI**
**Model I**	**0.23**	≥	135	110	**94.8**	**89.1-98.1**	15.1	9.9-21.6	44.9	38.6-51.4	80.0	61.4-92.3
		<	24	6								
	**0.61**	≥	8	41	35.3	26.7-44.8	**95.0**	**90.3-97.8**	83.7	70.3-92.7	66.8	60.3-72.9
		<	151	75								
**Model II**	**0.24**	≥	128	104	**95.4**	**89.6-98.5**	16.9	11.3-23.8	44.8	38.3-51.5	83.9	66.3-94.6
		<	26	5								
	**0.64**	≥	7	41	37.6	28.5-47.4	**95.5**	**90.7-95.2**	85.4	72.3-93.9	68.4	61.7-74.5
		<	147	68								
**Model III**	**0.25**	≥	132	104	**95.4**	**89.6-98.5**	14.3	9.2-20.8	44.1	37.6-50.7	81.5	61.9-93.7
		<	22	5								
	**0.63**	≥	7	4	36.7	27.7-46.5	**95.5**	**90.8-98.2**	85.1	71.7-93.8	68.1	61.4-74.2
		<	147	69								

Measures of accuracy were calculated for each of the individual diagnostic tests, and for the models on clinical background variables (model I), clinical background variables + sensitization to peanut extract in SPT or ImmunoCAP (model II), and clinical background variables + sensitization to peanut extract + sensitization to peanut components (model III). The three rows of threshold values given for each diagnostic test respectively indicate the cutoffs generally used in clinical practice, corresponding with a high sensitivity (closest to 95%), and corresponding with a high specificity (closest to 95%). **Bold** indicates the sensitivity and specificity estimates closest to 95%. *CI, confidence interval; CRD, component-resolved diagnostics; NPV, negative predictive value; PPV, positive predictive value; SPT, skin prick test.*

Table S7. Characteristics of subjects who underwent DBPCFC or had severe anaphylaxis to peanut

	**No or mild-to-moderate symptoms (N=47)**	**Severe symptoms (N=44)**	**p**
**Demographics**			
Age in years, *mean (±SD)*	26.0 (±9.9)	20.6 (±9.8)	0.013
Age < 14 years	6/47 (12.8)	11/44 (25.0)	0.180
Female sex	26/47 (55.3)	28/44 (63.6)	0.553
**Clinical background**			
Age at onset of symptoms < 14 years	25/47 (53.2)	35/44 (79.5)	**0.015**
Symptoms upon skin contact with peanut	7/45 (15.6)	17/30 (56.7)	**<0.001***
Family history of atopic disease	22/47 (46.8)	35/44 (79.5)	**0.002**
Atopic dermatitis	15/47 (31.9)	25/44 (56.8)	0.029
Birch pollen allergy‡	16/46 (34.8)	12/43 (27.9)	0.639
Grass pollen allergy‡	27/46 (58.7)	26/43 (60.5)	1.000
Mugwort pollen allergy‡	2/46 (4.3)	6/43 (14.0)	0.149
House dust mite allergy‡	21/43 (48.8)	30/43 (69.8)	0.079
Latex allergy‡	3/43 (7.0)	4/42 (9.5)	0.713
Cat/dog sensitisation ‡	31/47 (66.0)	34/43 (79.1)	0.249
**Peanut sensitisation§**			
**SPT peanut extract**			
Positive	37/46 (80.4)	36/42 (85.7)	0.708
Allergen/histamine wheal ratio, *median (IQR)*	0.92 (0.58-1.55)	1.28 (0.92-2.13)	0.238
**ImmunoCAP peanut extract**			
Positive	37/47 (78.7)	39/41 (95.1)	**0.031**
IgE level, *median (IQR)*	1.33 (0.51-6.17)	5.67 (1.54-57.47)	**0.031**
**Microarray peanut allergens**			
**Ara h 1**			
Positive	11/39 (28.2)	24/40 (60.0)	**0.009**
IgE level, *median (IQR)*	0.00 (0.00-0.32)	0.60 (0.00-5.6)	0.059
**Ara h 2/6**			
Positive	10/39 (25.6)	25/40 (62.5)	**0.002**
IgE level, *median (IQR)*	0.00 (0.00-0.24)	6.28 (0.00-19.34)	**0.014**
**Ara h 3/3.02**			
Positive	6/39 (15.4)	22/40 (55.0)	**<0.001***
IgE level, *median (IQR)*	0.00 (0.00-0.00)	0.44 (0.00-2.68)	0.088
**Ara h 8**			
Positive	18/39 (46.2)	10/40 (25.0)	0.084
IgE level, *median (IQR)*	0.00 (0.00-0.40)	0.00 (0.00-0.29)	0.243

Subjects with severe symptoms during DBPCFC (N=1) or life-threatening anaphylaxis based on patient history (N=43) were classified as severe. All measurements are in n/N (%) unless otherwise specified. P-values indicate difference between patients with no or mild-to-moderate and patients with severe symptoms to peanut. **Bold** indicates p<0.05. *Differences remained significant after Bonferroni correction. ‡Reported symptoms + matching sensitisation by SPT or ImmunoCAP. §Not all patients had complete testing for peanut sensitisation. *IQR, interquartile range; SPT, skin prick test.*

Figure S1. Occurrence of severe probable peanut allergy in European cities

Figure S1 Legend: -
