# Supplementary figures and images for: Estimating the Risk of Severe Peanut Allergy Using Clinical Background and IgE Sensitization Profiles
Source: Front Allergy. 2021 Jun 7;2:670789. doi: 10.3389/falgy.2021.670789 (PMC8974676; doi:10.3389/falgy.2021.670789)

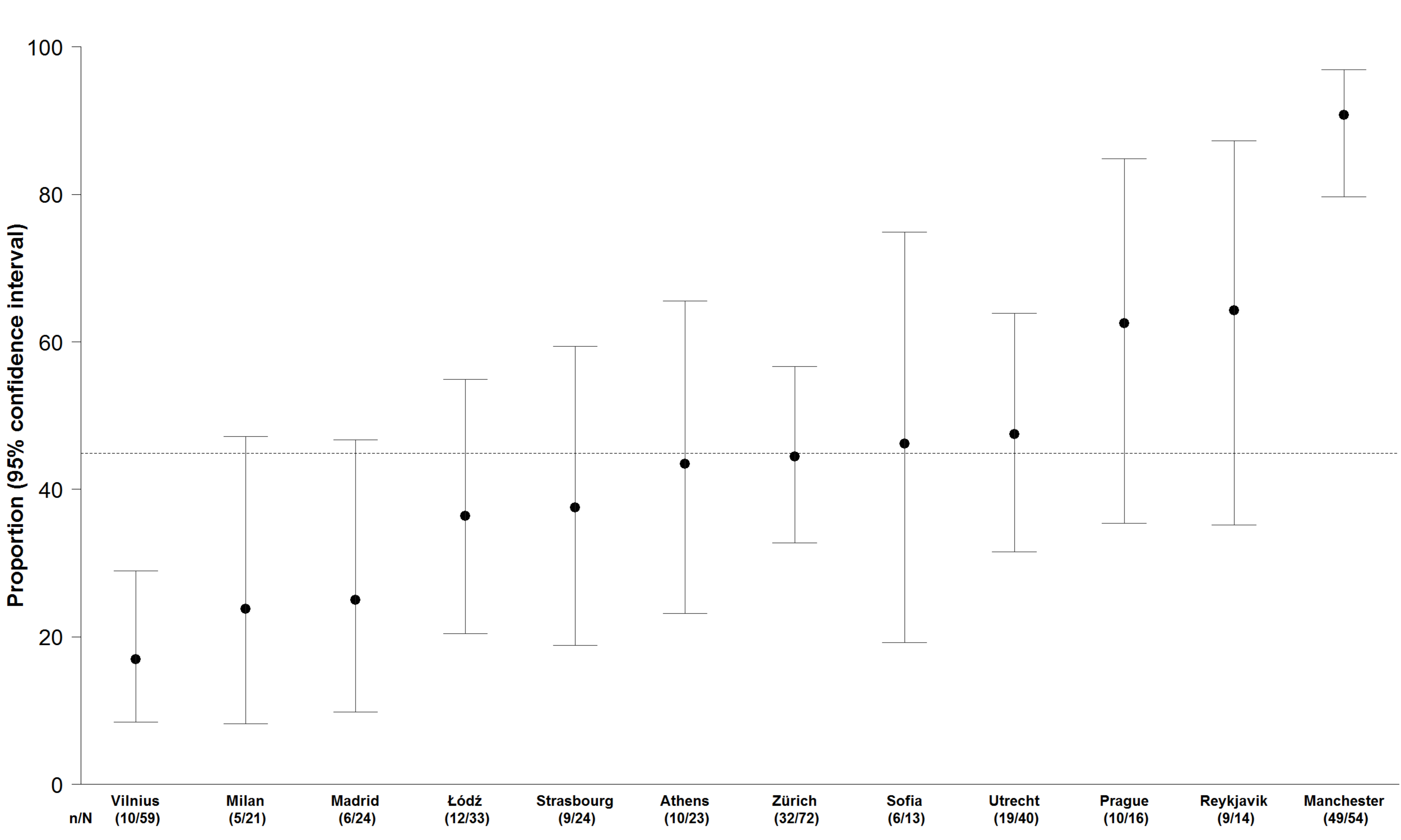

Supplement: Supplementary file 2 [file Image_1.pdf]
